# Supplementary material for: Causal Relationship between Aging and Anorexia Nervosa: A White-Matter-Microstructure-Mediated Mendelian Randomization Analysis
Source: Biomedicines. 2024 Aug 16;12(8):1874. doi: 10.3390/biomedicines12081874 (PMC11351342; doi:10.3390/biomedicines12081874)
Supplement: Supplementary file 1 [file biomedicines-12-01874-s001.zip › Supplementary Figure/Figure S4/Singe SNP plot of MR analysis for TL to ubm-b-1736.pdf]

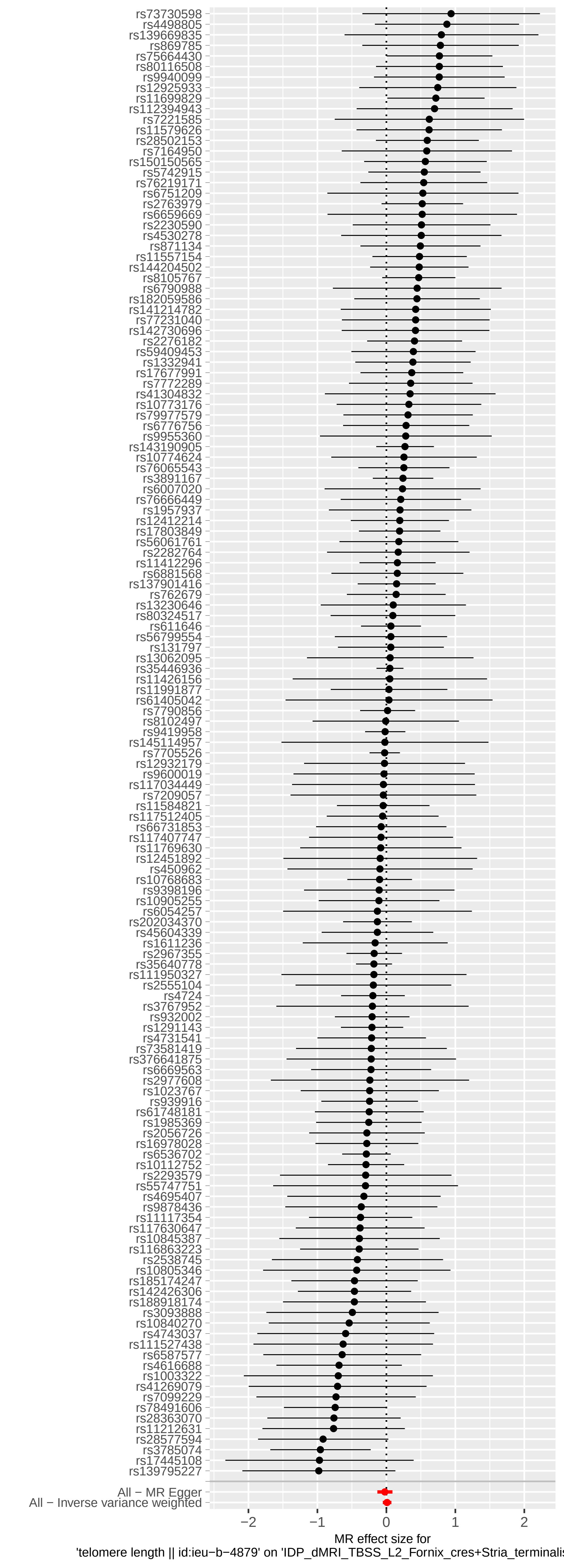
rs73730598
rs4498805
rs139669835
rs869785
rs75664430
rs80116508
rs9940099
rs12925933
rs11699829
rs112394943
rs7221585
rs11579626
rs28502153
rs7164950
rs150150565
rs5742915
rs76219171
rs6751209
rs2763979
rs6659669
rs2230590
rs4530278
rs871134
rs11557154
rs144204502
rs8105767
rs6790988
rs182059586
rs141214782
rs77231040
rs142730696
rs2276182
rs59409453
rs1332941
rs17677991
rs7772289
rs41304832
rs10773176
rs79977579
rs6776756
rs9955360
rs143190905
rs10774624
rs76065543
rs3891167
rs6007020
rs76666449
rs1957937
rs12412214
rs17803849
rs56061761
rs2282764
rs11412296
rs6881568
rs137901416
rs762679
rs13230646
rs80324517
rs611646
rs56799554
rs131797
rs13062095
rs35446936
rs11426156
rs11991877
rs61405042
rs7790856
rs8102497
rs9419958
rs145114957
rs7705526
rs12932179
rs9600019
rs117034449
rs7209057
rs11584821
rs117512405
rs66731853
rs117407747
rs11769630
rs12451892
rs450962
rs10768683
rs9398196
rs10905255
rs6054257
rs202034370
rs45604339
rs1611236
rs2967355
rs35640778
rs111950327
rs2555104
rs4724
rs3767952
rs932002
rs1291143
rs4731541
rs73581419
rs376641875
rs6669563
rs2977608
rs1023767
rs939916
rs61748181
rs1985369
rs2056726
rs16978028
rs6536702
rs10112752
rs2293579
rs55747751
rs4695407
rs9878436
rs11117354
rs117630647
rs10845387
rs116863223
rs2538745
rs10805346
rs185174247
rs142426306
rs188918174
rs3093888
rs10840270
rs4743037
rs111527438
rs6587577
rs4616688
rs1003322
rs41269079
rs7099229
rs78491606
rs28363070
rs11212631
rs28577594
rs3785074
rs17445108
rs139795227
All − MR Egger
All − Inverse variance weighted
−2
−1
0
1
2
MR effect size for
'telomere length || id:ieu−b−4879' on 'IDP_dMRI_TBSS_L2_Fornix_cres+Stria_terminalis
